# PET/CT-Based Dosimetry in ^90^Y-Microsphere Selective Internal Radiation Therapy: Single Cohort Comparison With Pretreatment Planning on ^99m^Tc-MAA Imaging and Correlation With Treatment Efficacy

**DOI:** 10.1097/MD.0000000000000945

**Published:** 2015-06-12

**Authors:** Yoo Sung Song, Jin Chul Paeng, Hyo-Cheol Kim, Jin Wook Chung, Gi Jeong Cheon, June-Key Chung, Dong Soo Lee, Keon Wook Kang

**Affiliations:** From the Department of Nuclear Medicine, Seoul National University Hospital (YSS, JCP, GJC, J-KC, DSL, KWK); Department of Nuclear Medicine, Seoul National University Bundang Hospital (YSS); and Department of Radiology, Seoul National University Hospital, Seoul, Korea (H-CK, JWC)

## Abstract

^90^Y PET/CT can be acquired after ^90^Y-microsphere selective radiation internal therapy (SIRT) to describe radioactivity distribution. We performed dosimetry using ^90^Y-microsphere PET/CT data to evaluate treatment efficacy and appropriateness of activity planning from ^99m^Tc-MAA scan and SPECT/CT.

Twenty-three patients with liver malignancy were included in the study. ^99m^Tc-MAA was injected during planning angiography and whole body ^99m^Tc-MAA scan and liver SPECT/CT were acquired. After SIRT using ^90^Y-resin microsphere, ^90^Y-microsphere PET/CT was acquired. A partition model (PM) using 4 compartments (tumor, intarget normal liver, out-target normal liver, and lung) was adopted, and absorbed dose to each compartment was calculated based on measurements from ^99m^Tc-MAA SPECT/CT and ^90^Y-microsphere PET/CT, respectively, to be compared with each other. Progression-free survival (PFS) was evaluated in terms of tumor absorbed doses calculated by ^99m^Tc-MAA SPECT/CT and ^90^Y-microsphere PET/CT results.

Lung shunt fraction was overestimated on ^99m^Tc-MAA scan compared with ^90^Y-microsphere PET/CT (0.060 ± 0.037 vs. 0.018 ± 0.026, *P* < 0.01). Tumor absorbed dose exhibited a close correlation between the results from ^99m^Tc-MAA SPECT/CT and ^90^Y-microsphere PET/CT (*r* = 0.64, *P* < 0.01), although the result from ^99m^Tc-MAA SPECT/CT was significantly lower than that from ^90^Y-microsphere PET/CT (135.4 ± 64.2 Gy vs. 185.0 ± 87.8 Gy, *P* < 0.01). Absorbed dose to in-target normal liver was overestimated on ^99m^Tc-MAA SPECT/CT compared with PET/CT (62.6 ± 38.2 Gy vs. 45.2 ± 32.0 Gy, *P* = 0.02). Absorbed dose to out-target normal liver did not differ between ^99m^Tc-MAA SPECT/CT and ^90^Y-microsphere PET/CT (*P* = 0.49). Patients with tumor absorbed dose >200 Gy on ^90^Y-microsphere PET/CT had longer PFS than those with tumor absorbed dose ≤200 Gy (286 ± 56 days vs. 92 ± 20 days, *P* = 0.046). Tumor absorbed dose calculated by ^99m^Tc-MAA SPECT/CT was not a significant predictor for PFS.

Activity planning based on ^99m^Tc-MAA scan and SPECT/CT can be effectively used as a conservative method. Post-SIRT dosimetry based on ^90^Y-microsphere PET/CT is an effective method to predict treatment efficacy.

## INTRODUCTION

Selective internal radiation therapy (SIRT) using ^90^Y-microsphere is an effective treatment option for treating inoperable hepatic malignancy. Many studies have reported therapeutic efficacy of ^90^Y-microsphere SIRT,^1–3^ with various treatment response rates in hepatocellular carcinoma,^4,5^ and metastasis from colorectal cancer^6^ or neuroendocrine tumors.^7^ Because hepatic malignancy is supplied with blood mostly from the hepatic artery,^8^^90^Y-microsphere can be selectively delivered to a target lesion by angiographic intervention. With a half-life of 2.67 days, ^90^Y emits β-particles with an average energy of 0.927 MeV. The average penetration range of the β-particles in tissue is 2.5 mm and 90% of the energy is absorbed within a sphere with a radius of 5.3 mm.^9,10^ This range is effective to deliver a large absorbed dose to a tumor while minimizing radiation hazard to the normal liver parenchyma, in case selective injection of ^90^Y-microspheres is successful.

Most crucial adverse effects in SIRT are related to radiation injury of the normal organs. Intrahepatic arterial shunt and consequent leakage of ^90^Y-microsphere to the lungs may cause radiation pneumonitis.^11^ Radiation injury of the normal liver may cause radioembolization-induced liver disease (REILD),^12^ which exhibits extensive sinusoidal congestion, hepatic atrophy, necrosis, and intimal fibrosis. To prevent these adverse effects and to determine activity requirement for treatment, planning angiography is performed before SIRT, in which ^99m^Tc-macroaggregated albumin (MAA) is injected into the target artery. Therapeutic efficacy and REILD are closely related to the injection activity of ^90^Y-microsphere,^12,13^ and thus, several methods such as the empirical method, the body surface area (BSA) method, and the partition model (PM) method have been suggested for activity planning.^10,14^ Among those methods, the PM method based on ^99m^Tc-MAA imaging is the most personalized one with a lower incidence of adverse effects,^13,15^ although some controversy exists regarding distribution equivalency between ^99m^Tc-MAA and ^90^Y-resin microsphere.^16^ Recently, single photon emission computed tomography (SPECT)/computed tomography (CT) has been widely used in clinical practice, with advantage of correct localization and attenuation correction. ^99m^Tc-MAA SPECT/CT has also been reported to be effective in activity planning for ^90^Y glass microsphere SIRT.^17^

After SIRT, the real distribution of ^90^Y-microsphere can be evaluated using bremsstrahlung scan or SPECT. Additionally, although ^90^Y emits a very small amount of positron (0.003%), positron emission tomography (PET)/CT for ^90^Y is also available with recent PET scanners, which are highly sensitive by adopting time-of-flight (TOF) algorithms.^18–20^ In this study, we performed activity planning for SIRT using ^90^Y-resin microsphere based on ^99m^Tc-MAA scan and SPECT/CT. The appropriateness of the ^99m^Tc-MAA imaging-based activity planning was evaluated in comparison with dosimetry by post-SIRT ^90^Y-microsphere PET/CT. Additionally, the efficacy of PET/CT-based dosimetry was also evaluated in terms of patient outcome. To the best of our knowledge, this is the first study applying ^90^Y-microsphere PET/CT to validate ^99m^Tc-MAA imaging-based activity planning.

## METHODS

### Patients and Study Protocol

Patients who were candidates for SIRT were included in this study consecutively. Candidates visited Seoul National University Hospital, Seoul, Korea, from June 2012 to August 2014. Contrast-enhanced CT was performed in every patient for pretreatment evaluation. SIRT was considered for patients with a life expectancy of more than 3 months and a malignant hepatic tumor that was unresectable and inadequate for chemotherapy due to its size.

Patients underwent treatment-planning angiography combined with ^99m^Tc-MAA injection. Patients with lung shunt fractions (LSF) superior to 0.2 were excluded from SIRT. Activity of ^90^Y-microsphere was planned based on ^99m^Tc-MAA SPECT/CT. SIRT was performed approximately 1 week after planning angiography by the same intervention radiologist. When injection sites were significantly altered (changes above major branch of hepatic artery level) between planning angiography and ^90^Y-microsphere SIRT, the patient was excluded from the analysis. Treatment response to SIRT was evaluated by the response evaluation criteria in solid tumors (RECIST) version 1.1 and progression-free survival (PFS) was evaluated. Follow-up contrast-enhanced CT or magnetic resonance imaging (MRI) was obtained approximately every 2 months after SIRT and when a patient presented abnormal symptoms, signs, or serum tumor marker increase.

### Planning Angiography and ^99m^Tc-MAA Imaging

Planning angiography and injection of ^99m^Tc-MAA (185 MBq) was performed by 1 interventional radiologist (H.C.K.), according to the previously published guidelines.^21^^99m^Tc-MAA was injected into the supplying arteries split according to the approximate volume ratio of tumor proportion supplied by each artery, in case there were more than 2 tumor-feeding arteries for a tumor. After the injection of ^99m^Tc-MAA, whole body scan and SPECT/CT were performed using a hybrid scanner combining a dual-head gamma camera and a 16-slice CT scanner (Discovery NM/CT 670, GE Healthcare, USA) equipped with low-energy high-resolution collimators. On whole body scan, conjugate anterior and posterior images were obtained over 10 minutes (table speed; 15 cm/minute) using 256 × 1024 matrices. SPECT images were acquired to cover the whole liver and the lower lung, by a step-and-shoot method (3° intervals) for 20 seconds per step. After SPECT acquisition, a helical CT scan was performed without using contrast agent and images were reconstructed into 3.75-mm slices. SPECT images were reconstructed on 128 × 128 matrices using an iterative algorithm (2 iterations, 10 subsets), including CT attenuation map-based attenuation correction, resolution recovery, and a postreconstruction Butterworth filter with a cut-off frequency 0.48 and order 5.

### Selective Internal Radiation Therapy and ^90^Y-Microsphere PET/CT

The planned activity of ^90^Y-labeled resin microsphere (SIR-Spheres, Sirtex Medical, Australia) was injected through a microcatheter according to the same method as the planning angiography. Injected activity was primarily determined using the PM method, further described below. However, in some patients with high LSF (>0.10), activity was determined by the BSA method and reduced by up to 40% according to the manufacturer's package insert.^22^

Immediately after completion of SIRT, PET/CT images were obtained for 1 bed position including the low chest and the upper abdomen, using a large field-of-view PET/CT scanner (Biograph mCT64, Siemens Healthcare, Germany). CT images were acquired first in a spiral mode (pitch 1.2, 120 kVp, and 35 mAs). PET images were acquired for 10 minutes using a 3D mode. CT images were reconstructed using a conventional filtered back projection method, 50-cm field of view, 3.0-mm postprocessing thickness and 2.0-mm increment per slice. PET images were reconstructed on 200 × 200 matrices using an iterative method including algorithms for point spread function recovery and TOF calculation (2 iterations, 21 subsets) with CT-based attenuation correction.

### Image Analysis for Activity Planning and Dosimetry

On ^99m^Tc-MAA whole body scans, regions of interest (ROIs) were drawn for the lungs and liver, and LSF was calculated by total counts in the lungs (TC_lung_) and the liver (TC_liver_) by Eq. (1) 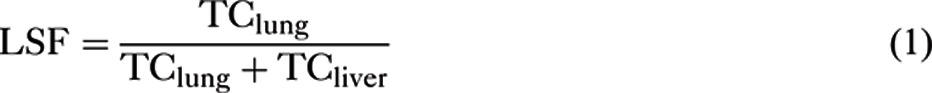


**FIGURE 1:**
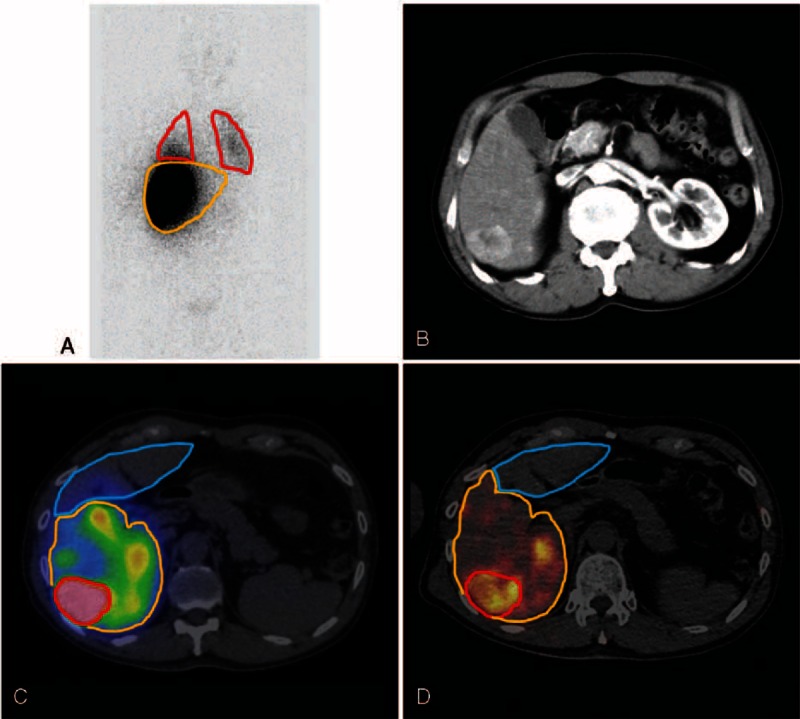
ROIs and VOIs for image analysis. LSF was measured on anterior and posterior ^99m^Tc-MAA planar scans. ROIs are drawn on the lungs (red) and liver (yellow) (A). With reference to contrast-enhanced CT images (B), VOIs for 3 partitions of tumor (red), in-target normal liver (yellow), and out-target normal liver (blue) were drawn on ^99m^Tc-MAA SPECT/CT (C) and ^90^Y-microsphere PET/CT (D).

For activity planning based on the PM method, 3 partitions were defined in the liver; tumor, in-target normal liver defined as the non-tumorous liver supplied by the target artery of SIRT, and out-target normal liver defined as the non-tumorous liver supplied by nontarget arteries of SIRT. On ^99m^Tc-MAA SPECT/CT images, ROIs of these 3 partitions were manually drawn on every slice on CT images of fusion SPECT/CT, with reference to contrast-enhanced CT images (Figure 1) using an analysis software package (Xeleris 3, GE Healthcare, USA). Volume rendering was done to acquire volumes of interest (VOIs) of the 3 partitions.

Mean uptake counts per unit volume in the tumor (*C*_tm_), in-target normal liver (*C*_in_), and out-target normal liver (*C*_out_) were measured in the VOIs, in addition to their volumes (*V*_tm_, *V*_in_, and *V*_out_). Absorbed dose of each partition was basically calculated by Eq(2). ^10,23,24^: 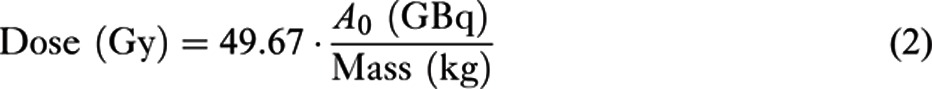


where *A*_0_ is the initial radioactivity in the partition. Tissue density was assumed to be 1.0 kg/L for the liver and tumor, and 0.3 kg/L^25^ for the lungs. For tumor, *A*_0_ was calculated by Eq.(3): 
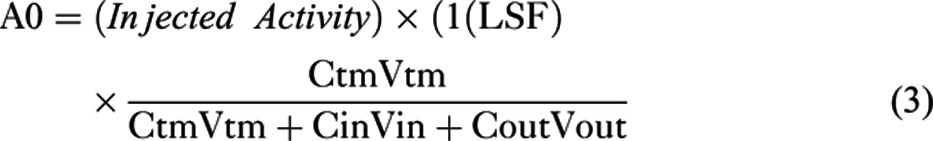


*A*_0_ for the in-target normal or out-target normal liver was also calculated using the same method. The activity of ^90^Y-microsphere was planned to meet the tolerance limits of the lungs (<20 Gy), and the in-target normal liver (<70 Gy), as well as the absorbed dose requirement for tumor treatment (>120 Gy).^26^ In some cases whose volumes of the in-target normal liver were considerably small, tolerance limit of the out-target normal liver (<30 Gy) was considered instead of that of the in-target normal liver.^27^ In addition to the PM method, recommended activity of ^90^Y-microsphere was also calculated by using the conventional BSA method,^3^ for comparison with the results of PM method.

Post-SIRT ^90^Y-microsphere PET/CT images were analyzed using a vendor-supplied software package (Syngo.via, Siemens Healthcare, Germany) (Figure 1). According to the same method as was used for ^99m^Tc-MAA SPECT/CT, VOIs were drawn for the lungs and 3 partitions in the liver for measuring mean uptake counts and volumes. Because PET/CT images were acquired for only 1 bed position, lung uptake was measured only in the scan-covered basal lungs, and total lung counts were presumed by multiplying lung volume and mean counts of the basal lungs included in PET/CT images. An average CT-based lung volume measurement value of 3.3 L was used. Absorbed dose to each partition was calculated from the measurements.

### Statistics

Paired Student *t* test and *χ*^2^ test were used to compare dosimetry parameters between the results from ^99m^Tc-MAA imaging and ^90^Y-microsphere PET/CT. Correlation between 2 parameters was evaluated using Pearson's correlation coefficient. For survival analysis, Kaplan–Meier survival analysis was used and PFS was compared between groups. In all statistical analyses, a commercial software package (MedCalc, Version 12.2.1.0, MedCalc Software, Belgium) was used and a *P*-value less than 0.05 was regarded as significant.

## RESULTS

### Patient Characteristics

^90^Y-microsphere SIRT was performed on 30 patients during the study period, among which 7 patients were excluded from the analysis because of major alterations in the injection sites between planning angiography and SIRT. Finally, 23 patients (M:F = 21:2, age 63.6 ± 12.4 years) were included in the analysis; 16 patients with hepatocellular carcinoma, 3 patients with cholangiocarcinoma, and 4 patients with metastatic liver mass from other cancers. Patient and tumor characteristics are described in Table 1. LSF was <10% in 19 patients, and 10–15% in 4 patients.

**TABLE 1:**
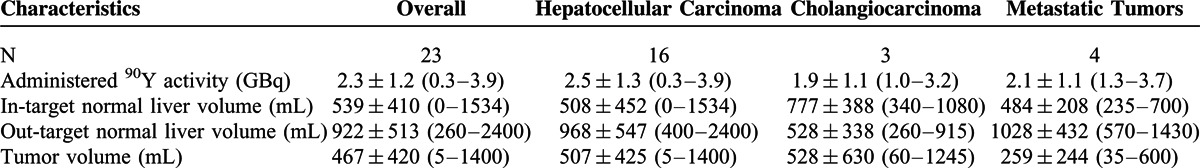
Patient and Tumor Characteristics According to Tumor Type

### Activity Planning

Injection activity was determined by PM method in 21 patients, and by BSA method in 2 patients with high LSF. In all patients, tolerance limits for the lungs were higher than absorbed dose requirement for tumor treatment. However, tolerance limit for out-target normal liver was slightly lower than absorbed dose requirement in 1 patient, and injection activity was reduced by 14 %. In 6 patients, the full planned activity was not injected because of arterial flow stasis and reflux (n = 4) or unexpected schedule change (n = 2). PFS was able to be evaluated in 22 patients, excluding 1 patient who was missing from follow-up. In activity planning by the PM method, absorbed dose requirement for tumor absorbed dose of 120 Gy was calculated as 2.1 ± 0.9 GBq (range 0.8–3.8 GBq), whereas it was calculated as 1.7 ± 0.2 GBq (range 1.1–2.1 GBq) from the BSA method. The absorbed dose requirement by the PM method was significantly higher than that by the BSA method (*P* = 0.02). From the PM method, the tolerance limits for the lungs and out-target normal liver were 31.3 ± 32.0 GBq (range 8.1–146.2 GBq) and 10.5 ± 10.2 GBq (range 1.1–42.8 GBq), respectively.

### SIRT and Post-SIRT Dosimetry

SIRT was performed 9 ± 5 days (range 1–15 days) after ^99m^Tc-MAA scan and SPECT/CT. All except 1 patient underwent SIRT more than 48 hours after ^99m^Tc-MAA injections to avoid possible embolization effect by MAA particles.^28^ The patient who underwent SIRT 1 day after ^99m^Tc-MAA injection did not exhibit any flow stasis or reflux during ^90^Y-microsphere injection. In SIRT, 2.3 ± 1.2 GBq (range 0.3–3.9 GBq) of ^90^Y-microsphere was injected. With these actual injected activities, the dosimetry results were calculated by the measurements on ^99m^Tc-MAA imaging and ^90^Y-microsphere PET/CT.

**FIGURE 2:**
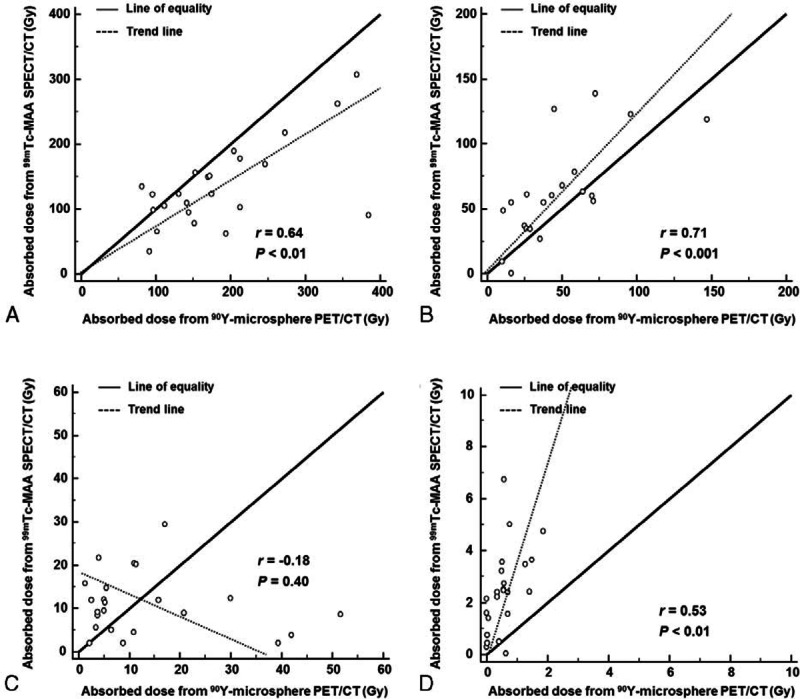
Scatter-plotting of mean absorbed dose of tumor (A), the in-target normal liver (B), the out-target normal liver (C), and the lungs (D) evaluated from ^99m^Tc-MAA SPECT/CT and ^90^Y-microsphere PET/CT.

Absorbed dose of each partition is shown in Table 2. LSF was evaluated to be lower by ^90^Y-microsphere PET/CT than by ^99m^Tc-MAA scan (*P* < 0.01). Absorbed dose of tumor was calculated lower by ^99m^Tc-MAA imaging (*P* < 0.01), but absorbed doses of the in-target normal liver (*P* = 0.02) and the lungs (*P* < 0.01) by ^99m^Tc-MAA imaging were higher than those by ^90^Y-microsphere PET/CT. There was no significant difference in absorbed dose of the out-target normal liver (*P* = 0.49). Significant correlations existed between the results by ^99m^Tc-MAA imaging and ^90^Y-microsphere PET/CT, in absorbed doses of tumor (*r* = 0.64, *P* < 0.01), the in-target normal liver (*r* = 0.71, *P* < 0.001), and the lungs (*r* = 0.53, *P* < 0.01), but not in absorbed dose of the out-target normal liver (*r* = −0.18, *P* = 0.40) (Figure 2).

**TABLE 2:**
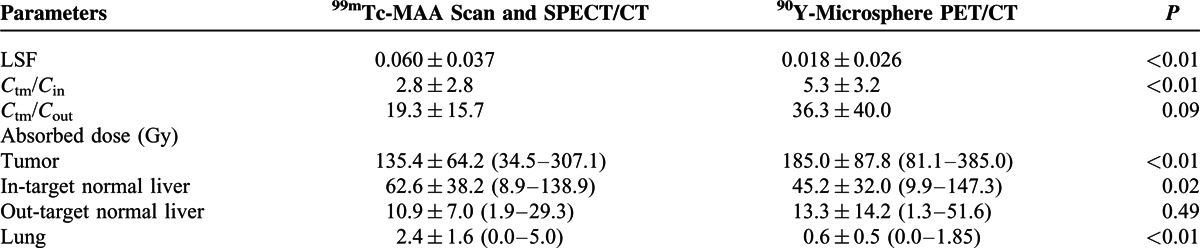
Dosimetry Results Evaluated by ^99m^Tc-MAA Imaging and ^90^Y-Microsphere PET/CT

### Correlation Between Tumor Outcome and Dosimetry

**FIGURE 3:**
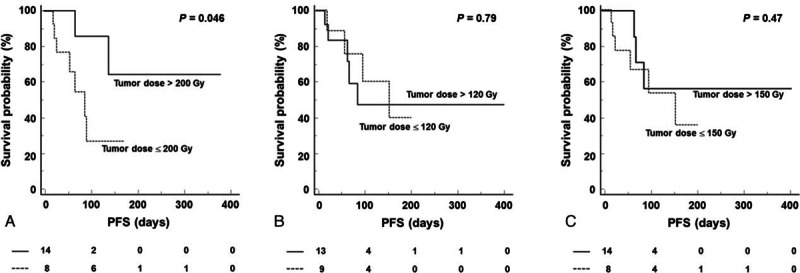
Kaplan–Meier survival curves for PFS according to mean absorbed dose of tumor, when patients were classified by tumor dose of 200 Gy from ^90^Y-microsphere PET/CT (A), and classified by tumor dose of 120 Gy (B) or 150 Gy (C) from ^99m^Tc-MAA SPECT/CT.

**FIGURE 4:**
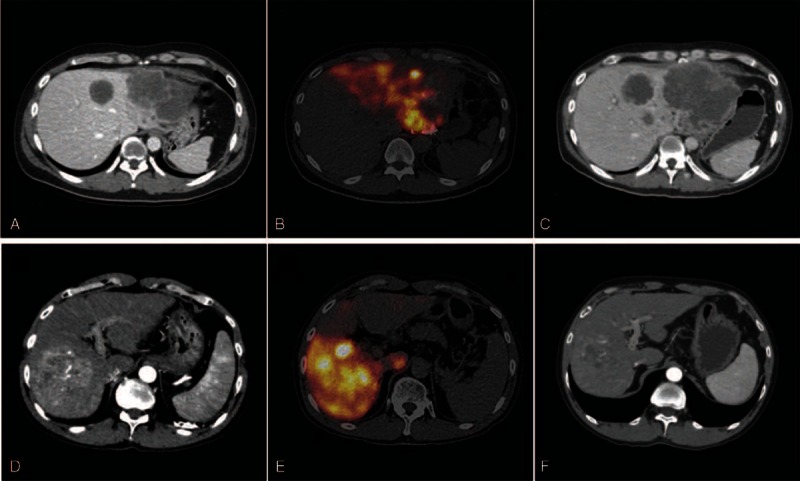
Representative cases. A 37-year-old male patient with hepatocellular carcinoma presented with a large mass in the left lobe with central necrosis on pre-SIRT contrast-enhanced CT (A). SIRT was performed and 2.3 GBq ^90^Y-microsphere was injected through the left hepatic artery. ^90^Y-microsphere PET/CT showed poor delivery of ^90^Y-microsphere to the central portion of the tumor (B). Tumor-absorbed dose was evaluated as 141.3 Gy. Post-SIRT CT showed enlargement of the tumor (C), and PFS was 21 days. Another 55-year-old male patient with hepatocellular carcinoma presented with a large mass in the right lobe on pre-SIRT CT (D). SIRT was performed through the right hepatic artery and 3.1 GBq ^90^Y-microsphere was injected (E). Tumor-absorbed dose was evaluated as 205.0 Gy. Post-SIRT CT showed shrinkage of the tumor (F) and PFS was 126 days.

SIRT was successfully performed in all patients without SIRT-related complication. Overall PFS of the patients was 207 ± 22 days. When patients were classified by tumor absorbed dose evaluated by ^90^Y-microsphere PET/CT, the groups exhibited a significant difference in PFS. In patients who had high absorbed dose (>200 Gy, n = 14), PFS was 286 ± 56 days (range 53–383 days), whereas it was 92 ± 20 days (range 18–170 days) in patients who had low absorbed dose (≤ 200 Gy, n = 8) (*P* = 0.046; Figure 3A). Between these 2 groups, there were no significant differences in other clinicopathologic factors that can affect PFS ^29–31^; tumor burden (%, *P* = 0.16), previous history of chemotherapy (*P* = 0.47), previous history of surgery (*P* = 0.23), extrahepatic metastases (*P* = 0.84), tumor type (*P* = 0.13), sex (*P* = 0.77), abnormal pre-SIRT total bilirubin (>1.3 mg/dL, *P* = 0.80), abnormal pre-SIRT serum albumin (<3.5 g/dL, *P* = 0.69), and poor performance status (ECOG grade >0, *P* = 0.84). Representative cases are shown in Figure 4.

According to tumor absorbed dose evaluated by ^99m^Tc-MAA SPECT/CT, only 3 patients received dose more than 200 Gy and statistical analysis was unavailable. When patients were classified by tumor absorbed dose of 120 Gy or 150 Gy evaluated by ^99m^Tc-MAA SPECT/CT, the 2 groups did not exhibit a significant difference in PFS (Figure 3B, C).

## DISCUSSION

In this study, we demonstrated that activity planning based on ^99m^Tc-MAA scan and SPECT/CT is closely related to post-SIRT dosimetry using ^90^Y-microsphere PET/CT, although there was a significant difference in the calculated values between the 2 imaging methods. By SPECT/CT, tolerance limits for the lung and liver were evaluated to be lower, and the absorbed dose requirement for tumor treatment was evaluated to be higher than by PET/CT, which results in narrower dose margin for activity planning. Thus, ^99m^Tc-MAA SPECT/CT can be used as a conservative activity planning method. Additionally, our study suggests that ^90^Y-microsphere PET/CT is an effective method for post-SIRT dosimetry and prediction of treatment efficacy.

In SIRT using ^90^Y-microsphere, post-SIRT dosimetry and efficacy has often been evaluated using ^90^Y bremsstrahlung scan or SPECT images.^16,32^ However, bremsstrahlung X-rays have a low count rate and a wide range of energy (50–250 keV) without an energy peak. Thus, the quality of bremsstrahlung imaging is still not satisfactory for accurate quantitative analysis, despite several attempts for optimizing reconstruction algorithm.^33,34^ Recently, PET imaging for ^90^Y has been available with PET scanners of enhanced sensitivity by adopting new reconstruction algorithms such as TOF calculation. Although the yield of positron emission from ^90^Y is very low, several previous studies have reported the feasibility of ^90^Y PET imaging and PET-based dosimetry.^35,36^ The activity distribution and counts on PET images are well correlated with the real activity measured in phantom studies.^36,37^ In the present study, we obtained ^90^Y-microsphere PET/CT images after SIRT and conducted dosimetry using the PM method, while previous studies performed voxel-wise dosimetry.^36,38,39^ Although the PM method does not provide information on heterogeneous dose distribution, it can be easily performed in clinical practice. Additionally, we defined 3 partitions in the liver by differentiating tumor, in-target normal liver, and out-target normal liver. Because radioactivity distribution is different between tumor and normal liver even if they are supplied by the same artery, this 3-partition model would provide more appropriate dosimetry results than a simple 2-partition model defining only tumor and normal liver.

In activity planning for ^90^Y-microsphere SIRT, simple methods such as empirical and BSA methods have a crucial limitation that individual condition of tumor size or arterial supply is not considered. PM methods are based on pretreatment ^99m^Tc-MAA imaging and can be a practical option for individualized planning. Because ^99m^Tc-MAA and ^90^Y resin microspheres theoretically do not redistribute after initial distribution, the effective half-life in a tissue is assumed identical to the physical half-life. Thus, dosimetry can easily be performed from the initial distribution of radioactivity. Recent application of SPECT/CT enables anatomical localization and more correct dosimetry compared with planar scan.

In the present study, significant differences existed between dosimetry results from pre-SIRT ^99m^Tc-MAA imaging and post-SIRT ^90^Y-microsphere PET/CT, for the lungs, in-target normal liver, and tumor. Despite the correlation, absolute values of the absorbed doses were overestimated for the lungs and in-target normal liver, whereas it was underestimated for the tumor by ^99m^Tc-MAA imaging, compared with PET/CT. There have been studies reporting discrepancy between ^99m^Tc-MAA and ^90^Y-microsphere imaging.^16,32,40^ The discrepancy was attributed to differences in particle distribution, caused by different particle features such as size, density, and injected amount.^16^ It also needs to be considered that catheter location and injection sites can be different between planning angiography and SIRT, even if an operator conducts the same procedures. Moreover, image characteristics of SPECT and PET such as resolution and sensitivity may be other causes for the difference. On ^99m^Tc-MAA scan and SPECT/CT, there is considerable spill-over effect from tumor to the lungs or adjacent normal liver. However, in spite of this limitation, ^99m^Tc-MAA SPECT/CT can be an effective tool for activity planning, because tolerance limit and absorbed dose requirement for tumor treatment evaluated on SPECT/CT may be regarded as relatively conservative values.

Additionally, tolerance limit in SIRT appears to be different from those in external beam radiation therapy, in which absorbed dose exceeding 30 Gy may result in radiation hepatitis.^27^ In our study, 4 patients received excess radiation to in-target normal liver (>70 Gy), 2 patients received excess radiation to out-target normal liver (>30 Gy), and 1 patient received excess radiation both to in-target and out-target normal liver. However, there was no significant hepatic complication or hepatic enzyme elevation in these patients (data not shown). In SIRT, several factors have been suggested as risk factors for REILD, including age, tumor type, tumor volume, and delivered activity.^12,13^ However, tolerance limit for normal liver has been different among different studies,^17,26^ and no single cutoff value has been used to prevent REILD. Further studies are required to determine optimal tolerance limit in ^90^Y-microsphere SIRT, based on accurate activity planning and dosimetry.

We adopted 120 Gy as the requirement for tumor treatment in planning. It is based on a previous study,^41^ in which radioactivity of a tumor was intraoperatively measured on the tissue surface using a beta probe. However, there is a considerable difference in absorbed doses between the central and peripheral portions of a tumor.^36,42^ Thus, radioactivity measurement on tissue surface using a beta probe may have underestimated the actual absorbed dose. In another study, tumor absorbed dose of 205 Gy in activity planning was suggested as a cutoff value for effective treatment.^17^ However, although it was reported that dosimetry based on ^99m^Tc-MAA SPECT/CT can be used to predict tumor response and survival in patients receiving ^90^Y-microsphere SIRT,^17^ tumor absorbed dose of 200 Gy measured on ^90^Y-microsphere PET/CT was a significant value to predict PFS, whereas tumor-absorbed dose of 120 Gy or 150 Gy in ^99m^Tc-MAA SPECT/CT-based dosimetry was not in our study. In spite of significant correlations between tumor-absorbed doses between measurements from ^99m^Tc-MAA SPECT/CT and ^90^Y-microsphere PET/CT, the dosimetry results based on ^99m^Tc-MAA SPECT/CT were not a significant prognostic factor probably due to some patients whose results were discrepant between the 2 imaging methods. Therefore, it is suggested that post-SIRT PET/CT scan for ^90^Y-microsphere is necessary for evaluating treatment efficacy and predicting prognosis. Further studies including more patients are required to determine optimal absorbed dose requirement for tumor treatment in ^90^Y-microsphere SIRT.

There are several limitations in our study. First, the study design was not prospective, and the injection sites were not exactly controlled between planning dosimetry and SIRT. Although we excluded cases in which injection sites were significantly altered, it can be a considerable limitation to compare the dosimetry between ^99m^Tc-MAA SPECT/CT and ^90^Y-microsphere PET/CT. Second, we evaluated pre-SIRT LSF on ^99m^Tc-MAA planar scan, because it covers whole lung field and is widely used in current clinical practice. However, attenuation or spill-over effect may have affected the results. In contrast, post-SIRT LSF was evaluated on ^90^Y-microsphere PET/CT that covers only the basal lungs. The differences in imaging method may have been a cause for the different dose results. Third, the enrolled case number was relatively small, despite statistical significance. Based on the present study, further studies including more cases are required to determine efficacy of post-SIRT ^90^Y-microsphere PET/CT and optimal absorbed dose requirement in SIRT.

## CONCLUSION

In this study, we demonstrated that activity planning based on ^99m^Tc-MAA SPECT/CT is closely related to the post-SIRT dosimetry based on ^90^Y-microsphere PET/CT, although there were still differences in the calculated doses from the 2 imaging methods. On SPECT/CT, the tolerance limit for the lung or liver was lower, and the absorbed dose requirement for tumor treatment was higher than on PET/CT, which results in narrower activity margin for effective and safe SIRT. Thus, ^99m^Tc-MAA SPECT/CT can be used as a conservative activity planning method. Additionally, our study suggests that post-SIRT ^90^Y-microsphere PET/CT is an effective dosimetry method and can be used to predict treatment efficacy.
